# Sensor-Based Wearable Systems for Monitoring Human Motion and Posture: A Review

**DOI:** 10.3390/s23229047

**Published:** 2023-11-08

**Authors:** Xinxin Huang, Yunan Xue, Shuyun Ren, Fei Wang

**Affiliations:** 1 Guangdong Modern Apparel Technology & Engineering Center, Guangdong University of Technology, Guangzhou 510075, China or hxx@gdut.edu.cn (X.H.); 2112117056@mail2.gdut.edu.cn (Y.X.); shirley.ren@gdut.edu.cn (S.R.); 2 Xiayi Lixing Research Institute of Textiles and Apparel, Shangqiu 476499, China; 3 School of Textile Materials and Engineering, Wuyi University, Jiangmen 529020, China

**Keywords:** wearable sensors, motion capture, posture recognition, wearable systems

## Abstract

In recent years, marked progress has been made in wearable technology for human motion and posture recognition in the areas of assisted training, medical health, VR/AR, etc. This paper systematically reviews the status quo of wearable sensing systems for human motion capture and posture recognition from three aspects, which are monitoring indicators, sensors, and system design. In particular, it summarizes the monitoring indicators closely related to human posture changes, such as trunk, joints, and limbs, and analyzes in detail the types, numbers, locations, installation methods, and advantages and disadvantages of sensors in different monitoring systems. Finally, it is concluded that future research in this area will emphasize monitoring accuracy, data security, wearing comfort, and durability. This review provides a reference for the future development of wearable sensing systems for human motion capture.

## 1. Introduction

With the continuous development of science and technology, people are paying more attention to exercise and health. Many researchers have turned their research into the human body, focusing on exploring the mystery of human movement. Human movement posture can generally be divided into gross and micro movements. Gross movements refer to large movements that require large muscle groups, which are the overall movements of the trunk and limbs, such as running, climbing, push-ups, and swimming. In contrast, micro-movements refer to small activities of small muscle groups, such as finger movements, vocal cord vibrations, and facial micro-expression changes. This paper will focus on the postural monitoring of human gross movements. 

**Figure 1:**
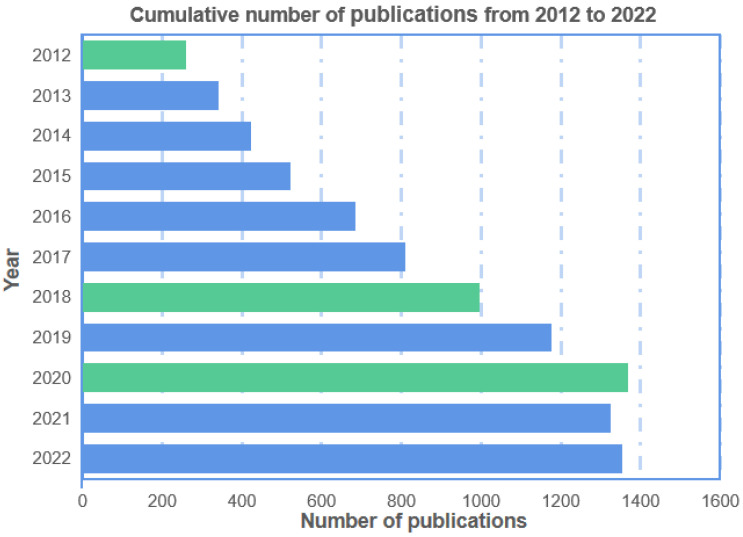
Number of papers related to human motion wearables published from 2012 to 2022. (Note: the statistics of publications come from ‘Google Scholar’, ‘Web of Science’, ‘CNKI’, and ‘Baidu Scholar’. The green color represents the key years).

The monitoring of human motion initially adopted the dynamic monitoring method of video image processing, that is, derive the human body’s dynamic characteristics and kinematic laws from the position and state of the human object at different times [1]. However, the data acquisition of cameras is liable to be affected by weather, light, distance, orientation, and other factors. Thus, it is challenging to perform high-precision recognition of motion poses. With the advancement of technologies, many sensors are applied to constructing wearable systems and monitoring human movement, in which sensors recognize human motion data, then process and analyze the data, and finally simulate the correct human motion posture. To date, research on human motion monitoring mainly concentrates on the development of sensor devices [2,3,4], as well as the design of wearable systems by integrating sensors into textiles or clothing. The system could achieve compatibility between functionality and comfort. In the past decade, the literature related to wearable devices for human motion monitoring has been increasing year by year, as shown in Figure 1. Since Jawbone launched the first sports bracelet in 2011, many entrepreneurs have invested in the smart wearable market, and 2012 was reckoned as the first year of intelligent wearable devices. Afterward, wearable devices became the hotspot in the information industry and attracted widespread attention. In 2018, studies on human motion wearables boomed, and almost 1000 research articles were published. Further in 2020, the related research ushered in another wave with 1369 papers published annually. It can be speculated that, as a part of smart wearables, wearables for monitoring human motion have already become a hot research area.

Due to the advantages of wearable sensors, including powerful computing, small size, and low cost [5], intelligent wearable devices are now used in various fields of human motion monitoring, the majority of which are medical and sports fields. In the medical field, motion monitoring is carried out in rehabilitation training, disease prevention and treatment, as well as movement disorder assessment. The wearables could assist doctors in evaluating the movement status of patients with Parkinson’s, amyotrophic lateral sclerosis, cerebral palsy, stroke, etc. Correspondingly, doctors could draw up a targeted rehabilitation plan and monitor the rehabilitation progress. Liang et al. [6] developed a motion capture device consisting of a wearable MEMS (Micro-Electro-Mechanical System) sensor and a mobile terminal, on which doctors can review the daily rehabilitation motion status of the patients in a remote manner and give timely guidance and advice. In the sports field, motion monitoring is commonly applied in exercise training and human kinematics studies. It can assist athletes to adjust their bodies to correct postures and rectify their movements in detail so as to develop a more scientific exercise plan and improve their performance. It can also be used in sports physiology research to help us understand the impact of sports on the body and its mechanisms. Moreover, intelligent wearable devices have excellent prospects in the areas of human-computer interaction, intelligent homes, industrial manufacturing, and virtual reality in the future [7,8,9].

To improve the comfortability of wearable devices, sensors are evolving from traditional rigid materials to emerging flexible materials. Previously, rigid materials dominated the wearable device market. Although their extensibility and compatibility are poor, the impact of rigid materials on wearing comfort is gradually reduced with the improvement of technologies such as Integrated Circuits (ICs) and MEMS (Micro-Electro-Mechanical Systems), which contribute to the miniaturization of sensor chips. In comparison, flexible materials present lightness, high extensibility, and a low loss of physical and chemical properties when stretched; therefore, wearable devices made of flexible materials have the advantages of lightness, good comfort, and a wide range of applications [10]. As a highly multidisciplinary technology, flexible electronics is promising, and many studies on flexible smart materials and sensors have increasingly emerged. By far, flexible sensors have begun to be commercialized. For example, Softcepter^TM^ fabric sensors developed by AdvanPro, HK, China, achieved mass production and were used in respiratory monitoring [11], muscle strength monitoring [12], and plantar pressure monitoring [13]. A fabric stretch sensor made by StretchSense, a company in Auckland, New Zealand [14], was adopted in the development of monitoring gloves to capture hand movements accurately. Tekscan^TM^, Boston, MA, USA, produced a flexible pressure measurement system that can monitor pressure distribution in the hand [15], lower extremities [16], and soles of the feet [17]. However, there are still various problems in practical applications of these flexible wearables, including poor durability, high production costs, low stability, and a lack of consistency. Hence, most motion monitoring sensors on the market are still made of rigid materials.

Up to now, wearable sensors applied to human motion monitoring have already been reviewed [18,19,20,21,22,23]. Nevertheless, there is a lack of analysis on the motion monitoring index system, a comprehensive comparison of rigid and flexible sensors, a summary of the wearable system design, as well as different angles of discussion on human motion parts, especially for gross motors involving trunk, joints, and limbs. Therefore, this paper, starting from the classification of human motions and focusing on three directions of human motion gesture monitoring index system, sensor characteristics, and system design, will comprehensively review the relevant literature on human motion gesture monitoring at home and abroad in recent years, summarize and analyze the current research status in this field, outline the limitations of current research, and predict its development direction in the future.

Note that certain physiological signals, including electromyography (EMG), are also closely related to human motion. However, compared with motion sensors, they are an indirect approach with lower precision for gross motor monitoring. For example, while EMG signals can provide muscle activity patterns in voltage and frequency, it is difficult to use these signals to obtain the exact motion amplitude or angle, especially in three-dimensional gross motor [24,25]. Therefore, this review excludes physiological signals.

## 2. Review of Literature

**Figure 2:**
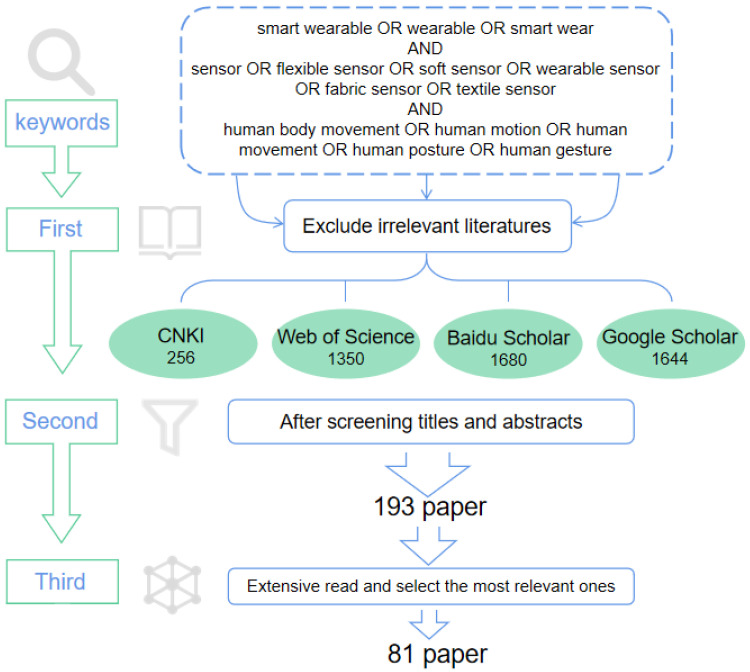
Flowchart of the literature screening process.

This study examines the latest developments in the technologies of sensor-based wearable systems for monitoring human movement posture. The literature review concentrated on sensors, monitoring indicators, and system design. To ensure a reliable literature collection process, four strategies were conducted step by step. Figure 2 presents the process of the literature review. Firstly, a systematic search of the related articles was carried out on databases such as ‘Google Scholar’, ‘Web of Science’, ‘CNKI’, ‘Baidu Scholar’, etc. Accordingly, the keywords and their combinations about wearable systems for human movement posture monitoring were summarized and modified based on our research purpose, which are (smart wearable OR wearable OR smart wear) AND (sensor OR flexible sensor OR soft sensor OR wearable sensor OR fabric sensor OR textile sensor) AND (human body movement OR human motion OR human movement OR human posture OR human gesture). After searching all the above keywords and excluding irrelevant papers, 4930 articles were obtained, which consist of 256 papers from the ‘CNKI’ database, 1350 papers from the ‘Web of Science’ database, 1680 papers from the ‘Baidu Scholar’ database, and 1644 papers from the ‘Google Scholar’ database. Thirdly, 193 relevant papers from the seared 4930 articles were confirmed by screening or reviewing their abstracts. Particularly, excluding the other 4737 papers followed two rules: (i) research on the movement of non-humans was excluded; (ii) research without the identification or monitoring of human movements and posture was excluded. Finally, 81 articles were selected for intensive reading after reviewing the 193 research papers extensively.

**Figure 3:**
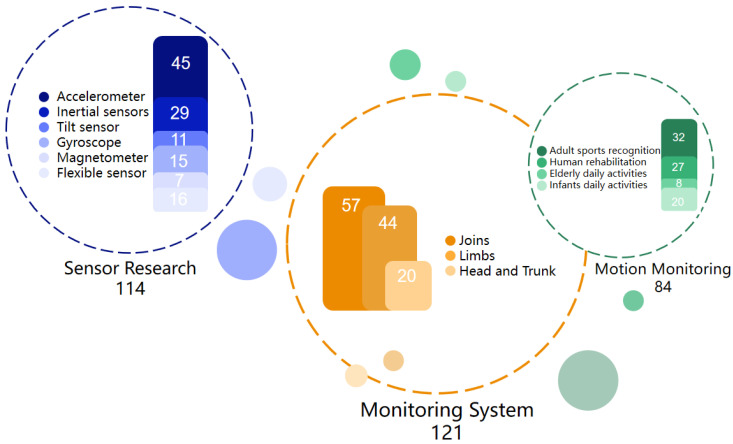
Overview of information sources.

Through a systematic literature search, 193 papers closely related to the sensor-based wearable system for monitoring human motion and posture were found. Figure 3 shows an overview of the literature sources. In particular, there were 114 papers related to human motion monitoring, among which 57 papers referred to limb motion monitoring, 44 papers referred to joint motion monitoring, and 20 papers referred to trunk motion monitoring. Most of the research concentrated on joint and limb movement. With respect to the topic of wearable system design, 84 research articles were collected, including 32 articles about the recognition of adult gross movement, 27 articles about human rehabilitation therapy monitoring, 8 articles about daily activity monitoring for the elderly, and 20 articles on daily activity monitoring for infants and children. Besides, there are 121 papers relating to sensor hardware research, including 45 articles concerning accelerometers, 29 articles concerning inertial sensors, 11 articles concerning tilt sensors, 15 articles concerning gyroscopes, and 7 articles concerning magnetometers. Regarding flexible sensors, most are applied to monitoring micro-movements. Only 16 related articles were obtained, in which flexible sensors were adopted for gross movement monitoring. However, flexible sensors are increasingly applied to the monitoring of joint activities, especially the identification of disease and rehabilitation treatment, which opens up an important application scenario for flexible wearable systems.

## 3. Monitoring Indicators of Human Motion

**Figure 4:**
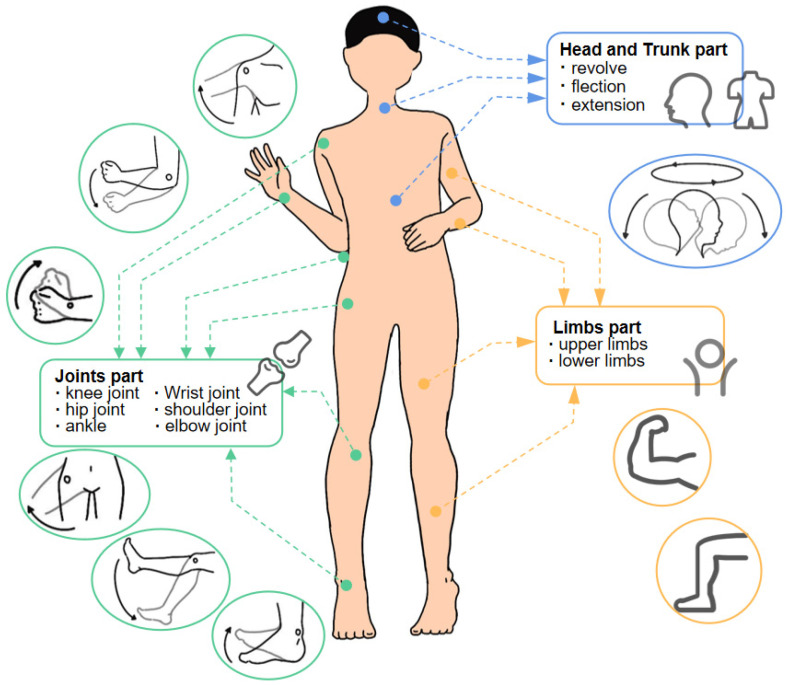
Schematic diagram of the monitoring area of human motion and posture.

The posture changes of human bodies can be decomposed into the changes of roll angle, heading angle, and pitch angle of the trunk, head, and limbs [6], as shown in Figure 4. Posture monitoring is essential for gait analysis, patient rehabilitation, and fitness activities. Among them, joint, limb, and trunk motion monitoring closely relate to human motion and posture monitoring.

### 3.1. Head and Trunk

**Figure 5:**
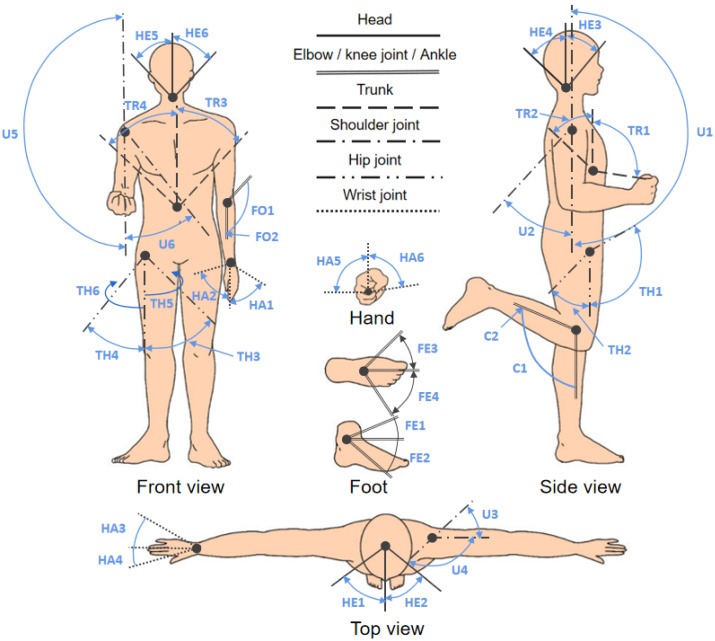
Range of motion for human joints. Reproduced with permission [39]. Copyright 2012, IEEE.

Compared with legs and arms, both the head and trunk present smaller amplitudes of movements, as shown in Figure 5. The angles and amplitudes of movements of the head and trunk were described in detail in Table 1. The head activities mainly contain levorotation and dextrorotation (±55°), hyperextension and flexion (−50° to +40°), as well as left and right lateral flexion (±40°). Particularly, the stress is primarily put on the neck during head movements. The trunk activities include flexion (+100°), hyperextension (−50°), and left and right lateral flexion (±50°); in particular, the stress is mainly exerted on the lumbar spine [26]. Monitoring of head and trunk movement is usually used for activity posture analysis, performance assessment, and rehabilitation [27]. Geng et al. [28] used inertial sensors worn on the chest, mid-forehead, wrist, and ankle to classify firefighter movements into standing, walking, running, lying, climbing, running upstairs, etc., to build applications such as the survival systems of first aiders or intelligent healthcare systems.

### 3.2. Joints

Human joints include the shoulder, elbow, wrist, hip, knee, and ankle joints (Figure 5). Joint movements refer to changes in joint angles and directions. These include flexion, extension, adduction, abduction, internal rotation, external rotation, anterior rotation, posterior rotation, inversion, and valgus. In particular, the wrist joints, elbow joints, and knee joints have two degrees of freedom, while the shoulder joints, hip joints, and ankle joints have three degrees of freedom and a more comprehensive range of motion. As shown in Table 1, the movements of the wrist joints contain extension (+65°), hyperextension (−75°), abduction (−15°), adduction (+30°), external rotation (−90°), and internal rotation (+80°); the movements of the elbow joints include flexion and extension (0~+145°); the movements of the knee joints could be presented as flexion and extension (0~−135°); the shoulder joints could achieve various movements, such as flexion (+180°), extension (−45°), horizontal abduction (−40°), horizontal adduction (+140°), abduction (+180°), and adduction (−45°); the activities of hip joint include flexion (+120°), extension (−45°), abduction (−45°), adduction (+40°), internal rotation (+35°), and external rotation (−30°); finally, the movements of the ankle joints contain dorsiflexion (+20°), plantar flexion (−45°), adduction (+45°), abduction (−50°) [29]. Generally, the study on human joint movements concentrated on the upper half and lower half, respectively. The shoulder joints, elbow joints, and wrist joints [30,31,32] are located on the upper half of the body, while the hip joints, knee joints, and ankle joints are located on the lower half of the body [30,33,34,35,36]. Up to now, more research has been conducted on upper-body joint activities.

The range of joint motion is correlated with age, gender, and other factors in humans, and it tends to decline gradually with increasing age, especially the knee joints [37]. In addition, injuries during activities and other related problems may lead to a decrease in the range of joint motion. Whereas, it is admitted that the range of joint movement possesses individual variability. Therefore, it is essential to research joint motion for the early clinical detection of joint problems or for determining the progress of joint rehabilitation. In sports training, joint motion signals were often used to identify deficiencies in athletes’ training and thus purposefully guide later training; in rehabilitation medicine, joint motion signals were employed to monitor the rehabilitation process of patients [38].

### 3.3. Limbs

The limbs are divided into two parts, e.g., the upper limbs and the lower limbs. The upper limbs include the upper arms, forearms, and hands, while the lower limbs refer to the thighs, calves, and feet. Most limb movements are caused by joint movements, which are usually evaluated in terms of movement direction and speed, including turning angle, rotation speed, bending, rotation, extension, flexion, etc. Particularly, the upper limbs can conduct movements like pushing, pulling, and whipping, while the lower limbs can conduct movements like cushioning, extension, and whipping [29]. Dynamic monitoring of limb movements can be applied in various fields, primarily in health care. Such as assessment of limb motor function, limb rehabilitation with electrical stimulation and other control modalities, differentiation of disease types, diagnosis, and treatment of motor dysfunctions [40]. The monitoring data of limb movements can also be used to construct 3D models to real-timely display the human body’s movements. Most of the limb-related research focused on monitoring lower-limb movements, such as gait monitoring.

## 4. Sensor Technologies for Human Motion Monitoring

Apart from the early video-based motion capture, the most widely used sensors for human motion capture include the traditional silicon-based sensors and the emerging flexible sensors. The former includes an accelerometer, gyroscope, magnetometer, inertial sensors, and tilt sensors; the latter involves soft strain and pressure sensors based on textile or polymer materials.

### 4.1. Traditional Silicon-Based Sensors

#### 4.1.1. Accelerometer

An accelerometer is a device that can measure the acceleration of an object. It is composed of a mass block, a damper, an elastic element, a sensitive element, an adaptation circuit, etc. According to the measurement principle, it can be divided into an angular accelerometer and a linear accelerometer. Accelerometers can detect deflections or stress deviations from external acceleration by means of their electrical signals [41]. Currently, the size of accelerometers is gradually decreasing [42] and becoming more wearable. They are widely used for fall recognition [43], gait monitoring [44], human motion analysis [45], elderly activity monitoring [46], children’s daily activity recognition [47], energy consumption estimation [48], and intelligent housing systems [45], etc. For example, timely recognition of the elderly falling to achieve appropriate protection [49]; being part of a smart housing system; forming an intelligent monitoring system as a part of a smart home system [45]; and assessing the type and intensity of daily activities, which is expected to improve energy consumption during exercise [48]. In recent studies, individual accelerometers have accurately distinguished abnormal motion [45,46,50]. However, reducing the number of sensors will decrease monitoring accuracy and make it difficult to identify more complex motions. Based on this, the design of monitoring systems that combine multiple accelerometers is being studied. The commercially available accelerometers commonly used in human motion monitoring are shown in Table 2. Particularly, the three-axis accelerometers in related studies are mainly from ADI and Freescale in the United States. These sensors are delicate and compact; the size is mostly 3~6 mm, and the thickness is not more than 1.5 mm. They have a wide measurement range, e.g., the accuracy is within ±2 g and the sampling rate is mostly within 50–100 Hz. In addition, in the current wearable system, triaxial accelerometers are often placed on the limbs and waist of the human body.

#### 4.1.2. Gyroscope

A gyroscope is a sensor that measures the angular variance or angular rate based on the Coriolis force [41]. It can measure the angular velocity of rotation at high speed and has a good dynamic response. Early researchers exploited a single gyroscope to measure angular acceleration. However, in order to measure the angular velocity of human motion, the accelerometer and gyroscope are often used together to decrease the error and improve the monitoring accuracy. Gyroscopes are now widely employed in electronic products such as laptops, smartphones, and navigation systems. However, long-term usage will cause it to be affected by random drift, resulting in drift errors. The commercial gyroscopes used mostly in human motion monitoring wearable systems are described in Table 3. The commonly used gyroscope is mainly from the Swiss STMicroelectronics and the United States Honeywell companies. These sensors are usually combined with accelerometers to form monitoring systems. Their assembled sizes range from 6.225 to 12,100 mm^3^, and the thickness is between 11 and 22 mm. Particularly, the ISM330DHCX sensor from STMicroelectronics is particularly compact, measuring less than 3 mm. These gyroscopes are available in a wide measurement range of up to 4000 dps, and the sampling rate is generally selected at 100 Hz. They are mainly used for angular acceleration monitoring of the upper limbs and trunk.

#### 4.1.3. Magnetometer

A magnetometer detects motion by using the earth’s magnetic field, and its measurement accuracy is high at static. It calculates the carrier orientation attitude by sensing the local magnetic field flux. Its indoor positioning results are often more accurate than GPS. When the earth’s magnetic field is constant, the magnetometer has good measurement performance at rest, and it does not drift over time. Therefore, it is often used in combination with accelerometers and gyroscopes to form an inertial measurement unit (IMU), which is used to correct the heading angle offset caused by gyroscope drift. Accordingly, the accuracy of attitude monitoring during human motion has improved.

#### 4.1.4. Inertial Sensors

Since accelerometers are susceptible to temperature and other factors, the accelerometers and gyroscopes or magnetometers are often integrated to form an inertial sensing unit ‘IMU’ to ensure monitoring accuracy, which is known as inertial sensors. Inertial sensors are increasingly used because of their small size, low cost, and ability to measure 3D knee angles with high accuracy. Besides, they are not affected by light, space, or other environmental factors. The sensors have been applied to recognizing gymnastic movements, non-invasively assessing knee function, and treating diseases such as Parkinson’s, etc. When monitoring human motion and posture, the monitoring error is mainly caused by soft tissue artifacts, sensor movement, and the relative motion of bones because the inertial sensors must be closely connected to the human body. The inertial sensors used in intelligent wearable systems are mostly nine-axis. As shown in Table 4, the commercial inertial sensors widely used are mainly produced by InvenSense, Bosch Sensortec, and Shimmer, Germany. InvenSense inertial sensors are the most popular. The size of this type of sensor is between 2.7 and 14.4 mm^3^, and its thickness is less than 1mm. It is primarily installed in the human chest, wrist, calf, thigh, and ankle joints.

#### 4.1.5. Tilt Sensor

**Figure 6:**
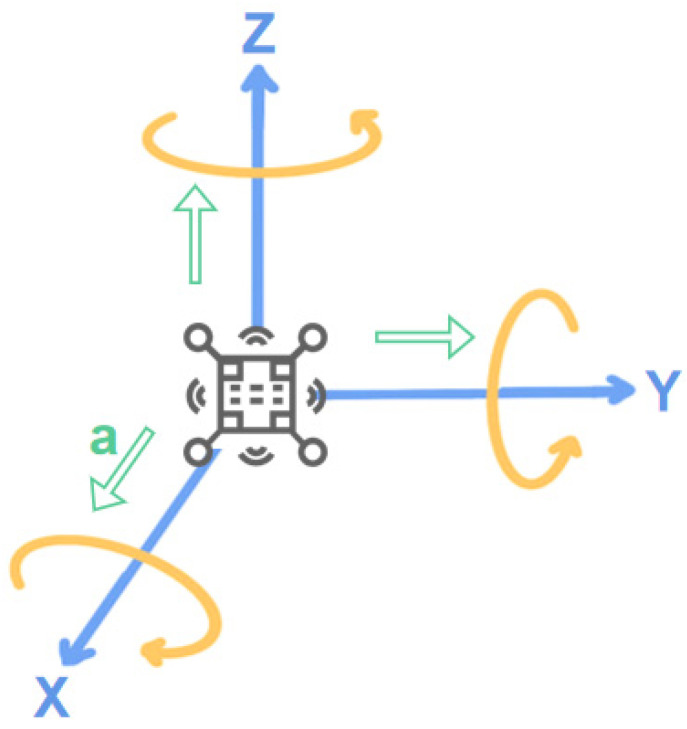
Inclination sensor measurement principle.

The inclination sensor is designed based on the principle of gravitational acceleration, which combines accelerometers and gyroscopes. As shown in Figure 6, the system’s inclination can be obtained by measuring the acceleration of the X, Y, and Z axes and further calculating the angle between each axis and the acceleration of gravity [56]. Inclination sensors are mostly used for lower limb posture monitoring [54,70,71]. For example, Cui et al. [54] designed a device to monitor human gait in which five inclination sensors were tied to the subject’s trunk, thigh, and calf to measure the angle with the horizontal plane. In the process of motion monitoring, the error of the inclination sensor is mainly ascribed to human factors and environmental factors. The human factors include the wearing position and installation method, and the environmental factors mainly contain temperature and surrounding noise. To solve this problem, Wang et al. [71] designed the signal processing of leg inclination sensors for footed robots. In this system, noise and temperature effects can be filtered out during measurement, and the error can be controlled within 0.75%.

### 4.2. Flexible Sensors

Flexible sensors have received a great deal of attention in the last few decades due to their excellent properties of high stretchability, biocompatibility, high compliance, and high sensitivity [72]. They are mainly used in human motion monitoring, human-computer interaction, telemedicine, and augmented reality/virtual reality. Several review articles [73,74,75] recommended flexible sensors, and they elaborated on the sensors from the perspective of sensing principles. According to the substrate materials, flexible sensors can be divided into two categories: textile sensors and thin-film sensors, both of which can include strain sensors and pressure sensors.

#### 4.2.1. Substrates of Flexible Sensors

**Figure 7:**
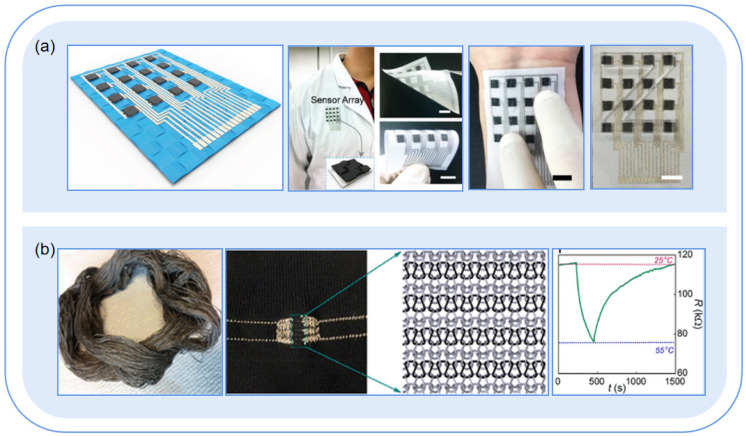
Combinations of flexible sensors and fabrics. (**a**) Coated on the fabric surface. Reproduced with permission [3]. Copyright 2017, John Wiley & Sons; (**b**) coated on fiber or yarn first, and then knitted into fabric. **Left**: Hank of rGO-dyed (coated) cotton yarn; **Middle**: Knitted temperature sensor with graphene-coated yarn; **Right**: Time response property of the knitted temperature sensor. Reproduced with permission [79]. Copyright 2019, American Chemical Society.

Textile sensors use fibers, yarns, and fabrics as substrates to enable various sensing functionalities through material improvement and structural design [76,77]. For example, Li et al. [78] developed a microfiber membrane with omnidirectional super elasticity (recovery from a 2000% strain), permeability (over 90% of cotton), and super hydrophobicity (water contact angle of 154.2°), which can identify materials and monitor hand posture. Textiles are one of the most ideal wearable materials, with both sensing characteristics and fabric properties such as excellent softness, durability, biocompatibility, and lightness. Textile sensors have undergone four generations of development. In the first and second generations, fabrics were used only as carriers. The rigid components of electronic devices were attached to or embedded in the textile. In the third generation of flexible fabric devices, flexible fabric devices and rigid microelectronic components formed a hybrid system by means of heterogeneous system integration technology, which contributed to the emerging flexible electronic components. The fourth generation used all electronic fabric devices to form an electronic system. At present, the third generation of hybrid systems has been successfully applied, and the research on the fourth generation of all-fabric electronic systems has made some achievements. However, the development still presents a bottleneck, mainly in the conflict between the functionality of electronic devices and the intrinsic properties of the fabric [72]. One typical example is the coated textile sensors, in which the sensing materials were directly coated on the fabric surface (Figure 7a). For example, Liu [3] developed a pressure sensor using nickel-coated polyester fabric and carbon nanotubes (CNT), which has the advantages of high sensitivity, good linearity, and wide range, but the lack of adhesion of the coating affects its washing performance. Another typical example is the textile-type structure, in which the sensing materials are coated onto yarns and fibers (Figure 7b), which are then woven into conductive lines by knitting, weaving, embroidery, or weaving processes. Afroj et al. [79] used rGO-coated cotton yarn to weave a temperature sensor via knitting, which is wear-resistant and has excellent deformability and washability. However, its conductivity is poor, and its sensitivity and response are average. Since the flexible system is different from its conventional rigid counterpart, new evaluation criteria and physical models are needed. Besides, the conductive yarns are prone to corrosion, leading to low wear resistance and low conductivity. Further research is still needed before commercialization.

Thin-film sensors are worn on the skin, with the concept of e-skin being widely known for designing thin-film sensors that can mimic the human skin. A typical example is from John A. Rogers’s research group [80]. They designed a fully elastomeric strain gauge directly attached to human skin that can be integrated with stretchable electronics. Such sensors can also be applied to treat swallowing difficulties caused by head and neck cancers, with thicknesses as low as 300 μm. They can accurately monitor the electrical signals of salivary swallowing [81], as well as the pressure monitoring of intracranial, intraocular, and intravascular cavities [82]. It can also be used for neonatal physiological signal monitoring, open (“orifice”) layout, and pre-curved design to reduce adhesion damage [83]. Thin-film sensors were initially limited to monitoring small deformations in the human body (fingers, wrists, etc.) [84,85]. Hydrogel sensors have recently been used to identify larger deformations (joints, neck, etc.) [86,87]. However, most e-skin sensors are continuous film-like structures that can only be stretched and bent in every direction, and these sensors do not allow for significant double curvature bending or in-plane shear [88]. This dramatically limits the strain forces that this type of sensor can withstand. As regards this limitation, Chen et al. [89] developed a hydrogel self-powered human motion sensor with an elongation at a break of 2800% and good strain sensitivity (GF = 4 at 200% strain). In addition, Gao et al. [90] reported a biological tissue inspired ultra-soft microfiber composite ultrathin (<5 μm) hydrogel film that can be seamlessly attached to various rough surfaces. The hydrogel has prominent mechanical strength (tensile stress~6 MPa) and anti-tearing properties.

The high conductivity and sensitivity of flexible sensors have long been the focus of research. Many scholars have integrated highly conductive nanomaterials with flexible materials. For example, Matsuhisa et al. [91] investigated the bonding process of silver particles on a fabric surface and demonstrated the feasibility of a fully printed sensor network suitable for complex and dynamic surfaces; Han et al. [92] designed a dual conductive percolation network hydrogel strain sensor; Yu et al. [93] reported an all-in-one, stretchable, and self-powered elastomer-based piezo-pressure sensor (ASPS); Zhang et al. [94] prepared a hydrogel composite containing MXene (Ti_3_C_2_T_X_) with a gauge factor (GF) of 25. Based on this, Bai et al. [85] found that the electrical conductivity of MXene/PAA hydrogels gradually increased from 8 to 69 mS m^−1^ with the increase of MXene.

#### 4.2.2. Flexible Strain and Pressure Sensors

Strain sensors can convert the degree of deformation of mechanical motion into electrical signals. They are available in three categories: resistance, capacitance, and voltage [72,95]. Among them, resistive and capacitive strain sensors are most widely used as they are simple and easy to use [96,97].

**Figure 8:**
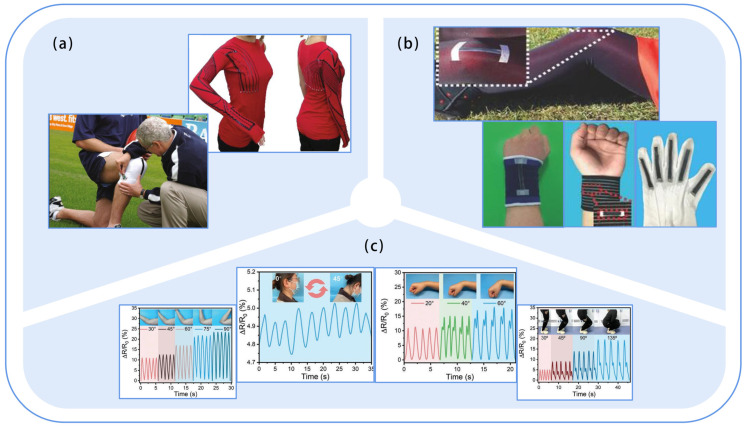
Application of flexible strain sensors for human motion monitoring. (**a**) Carbon-loaded elastomer-sensorized garment developed at the University of Pisa for kinesthetic monitoring. Reproduced with permission [105]. Copyright 2011, Springer Nature; (**b**) Strain sensors assembled on clothing or accessories to recognize small and large movements. Reproduced with permission [109]. Copyright 2016, John Wiley & Sons. (**c**) NSD-Gel electronic skin attached to the human neck, wrist, elbow, ankle, and knee for real-time motion monitoring. Reproduced with permission [72]. Copyright 2023, John Wiley & Sons.

Early studies of flexible strain sensors in human motion monitoring focused on fine movements, such as finger movements [95,98,99,100,101], facial expressions [85,102,103,104], and limb muscle contractions, etc. [12]. However, current research has been extended to body joints (wrist, ankle, neck, knee, elbow, etc.) [72,102,105,106] and body posture [107]. For example, Coyle S. from the University of Pisa [105] reported a sensor-printed garment for kinesthetic monitoring (Figure 8a), in which a composite material consisting of a silicone matrix and CB (carbon black) powder is printed on Lycra^®^/cotton fabric to provide real-time feedback on the wearer’s limb orientation. Besides, Zhang et al. [108] designed a rose-shaped strain sensor system consisting of three graphite/silk fiber (GSF, Graphene Silk Fiber) strain sensors, which were mounted on the wrist to monitor human motion in multiple directions. Subsequently, Wang et al. [109] developed a strain sensor with a woven structure (Figure 8b) that can monitor human motion postures, including joint movements, finger movements, wrist flexion, etc. The strain sensor could detect a strain of up to 500% and maintain long-term stability. Then, Bai et al. [72] prepared a transparent, mechanically robust, environmentally stable, and versatile natural skin-derived organic hydrogel (NSD-Gel) sensor as given in Figure 8c, which can recognize neck, wrist, elbow, ankle, and knee joint movements. In addition, Kong et al. [110] found that a strain sensor based on polyurethane (PU) yarn could measure up to 50% of strain stably at only 1% strain for more than 100,000 cycles. Bai et al. [85] developed an MXene/polyacrylic acid (PAA) hydrogel flexible strain sensor with high sensitivity (GF ~ 4.94), wide detection range (0–1081%), and stable output signal that can not only recognize subtle movements such as frowning, smiling, and swallowing, but also accurately monitor human motion with significant deformation.

**Figure 9:**
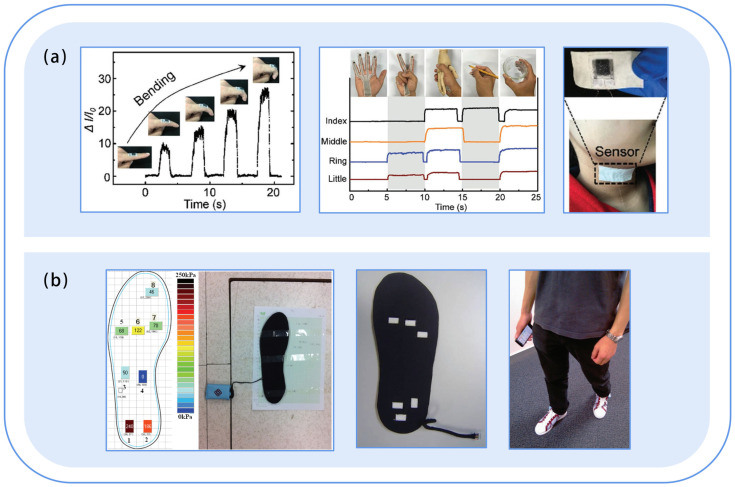
Study of flexible pressure sensors in human motion monitoring. (**a**) Textile sensors attached to the skin of a curved finger to monitor different mechanical forces in real-time. Reproduced with permission [3]. Copyright 2017, John Wiley & Sons; (**b**) Textile pressure sensor integrated with a shoe insole to monitor plantar pressure distribution and gait analysis [113].

Unlike strain sensors, pressure sensors convert pressure into an electrical signal [111]. Most current pressure sensors have a sensing range of more than 10 kPa and can be used for finger motion monitoring (Figure 9a), such as finger bending force, pressure, twisting, and extension force [3], as well as for human joint motion recognition. For example, in 2020, Zhu et al. [112] developed a novel highly shape-adaptive, self-powered piezo electronic skin that can be mounted coordinately on arbitrarily curved surfaces (e.g., twisted human skin) or moving surfaces (e.g., curved joints). However, it is currently mainly used for gait monitoring. In 2012, Shu [113] integrated it into a fabric insole to develop an intelligent wearable system (Figure 9b) for plantar pressure distribution measurement and gait analysis; Wang et al. [114] developed a textile capacitive pressure-sensing insole for gait pattern analysis; and Zhou et al. [115] developed a brilliant motion array with an 8 × 16 pressure sensing array that can monitor calf muscles during daily exercise and perform motion quality evaluation. In addition, pressure sensors are also widely used for early screening of diseases. For instance, Shu et al. [13] developed iShoe, a pair of shoes that can map foot pressure anytime and anywhere, and the system has been successfully tested in local hospitals in Hong Kong for the prognosis of diabetic foot syndrome. Cheng et al. [116] reported a piezoresistive pressure sensor based on PPy/rGO/FSF (nylon fabric-latex foam-nylon fabric (FSF), reduced graphene oxide (rGO), and in situ molded polypyrrole (PPy)) composite for Parkinson’s disease detection by monitoring the time of head lift and elevation. However, pressure sensors can also be applied to smart homes. Yu et al. [117] reported an all-in-one conformal pressure sensor (ACPS) that can be deployed in the human body and the room to care for the elderly living alone.

## 5. Design of Wearable Sensor Systems

Wearable sensors for human motion monitoring can be divided into two parts. One part monitors the micro-movements of the human body, such as fingers, facial five senses, throat, foot pressure, etc. The other part is the gross movements of the human body, including trunk monitoring, limb monitoring, and joint activity monitoring. The study of limb monitoring can be further divided into upper and lower limbs [6,42,63,67,118]. Upper and lower limb monitoring is normally used in the rehabilitation of Parkinson’s and other diseases, as well as fall prediction. In addition, research on the upper limb focuses on identifying limb activity. Research on joint activity focuses on the monitoring of knee joints [35,36,119], aiming to track and assess the function of the knee joint in a non-invasive manner for fall monitoring and prevention, athletic performance assessment, and rehabilitation progress observation.

**Figure 10:**
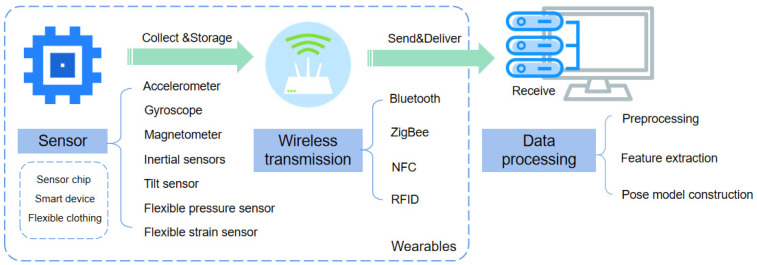
Framework of the sensor monitoring system.

The sensor monitoring system mainly comprises three modules: the sensor module, the wireless transmission module, and the data processing module. One specific monitoring system composition is shown in Figure 10, which consists of sensors for monitoring human movement, collecting and storing data, a wireless transmission device for transmitting the data, and finally an electronic receiving device that subsequently performs a series of processing on the data. The wearable device system design studied in this paper emphasizes sensor selection and wearable structural design.

### 5.1. Hardware Design of the Wearable System

**Figure 11:**
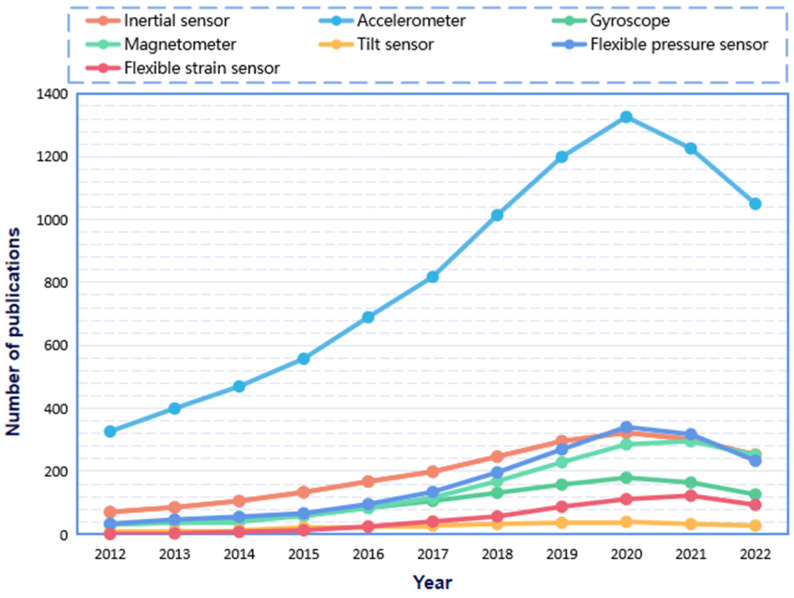
Trends in the use of wearable hardware sensors in human motion monitoring studies (based on the references considered in the review process).

The selection of sensors includes their type and quantity, as shown in Table 4. The identification and monitoring of falls and daily activities is usually accomplished by adopting one to three accelerometers. They are typically set near the thigh, waist, and wrist. Jala et al. [45] (Air University, Islamabad, Pakistan) used only one single accelerometer to recognize and monitor 14 daily activities, such as brushing teeth, combing hair, drinking water, pouring water, drinking soup, eating meat, walking up and down stairs, getting up, lying down, getting up, sitting down, and using a telephone. Additionally, more complex motion recognition often requires four to nine inertial sensors strapped to the limbs as well as to the waist and abdomen, etc. Cui et al. [54] reported that, to study exoskeleton gait, five MPU-6050 6-axis MEMS inertial sensors were tied to the abdomen, the upper one-third of the thigh, and the lower one-third of the calf. There are also studies employing more sensors for monitoring. Esfahani et al. [120] used 18 inertial sensors, which were fixed at the extremities (12), head (1), both shoulders (2), back (1), back waist (1), and forehead (1), aiming to study the Sharif Regno motor instrumentation system. It can be concluded that in the future, the choice of the number of wearable sensors will no longer be limited to a single one, but will tend to be a sensing system with multiple sensors working collectively in the form of a matrix. In addition, through statistical analysis of the types of sensors in the studies, it was found that, as shown in Figure 11, the status of accelerometers in human motion monitoring cannot be underestimated. As the research on magnetometers and gyroscopes gradually deepens, the research on the combined inertial sensors gradually heats up. More and more studies tend to use inertial sensors, which will become the focus of attention in motion sensing in the future, and more researchers will consider it the first choice for motion monitoring. In addition, research on flexible sensors composed of flexible materials is increasing rapidly and is mainly focused on the rehabilitation medical field. Representatively, Shu [113] found that a system of six sensors has been validated to monitor plantar pressure accurately. However, due to the lack of standards and the establishment of an evaluation system, the pace of its industrialization is more complex. Currently, most research still focuses on exploring conductive and flexible substrate materials to improve their conductivity and accuracy.

### 5.2. Structural Design of the Monitoring System

**Figure 12:**
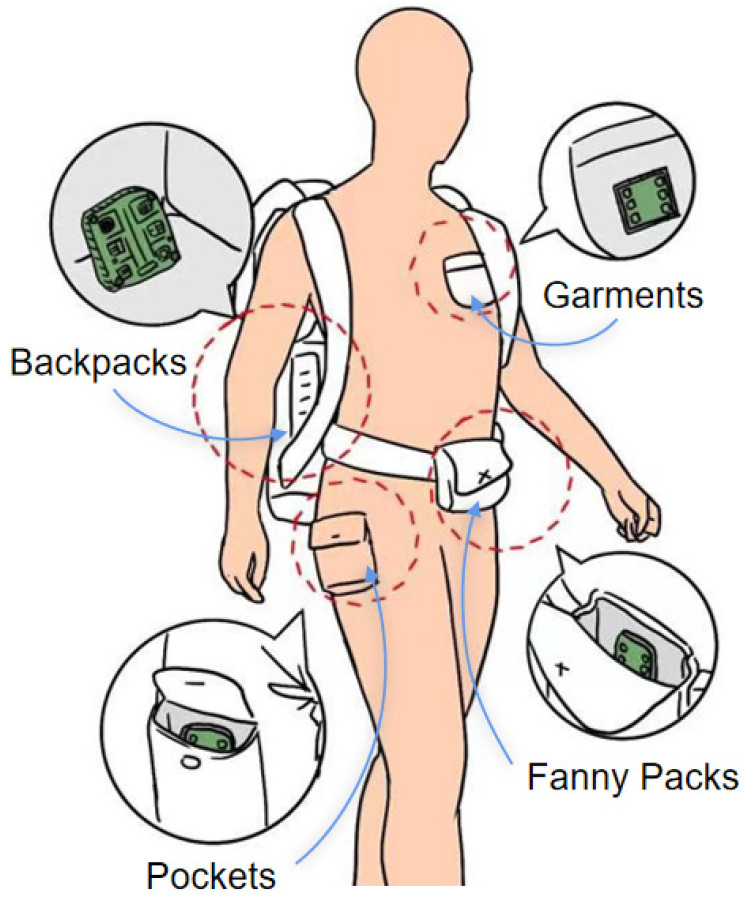
Early wearable forms.

**Figure 13:**
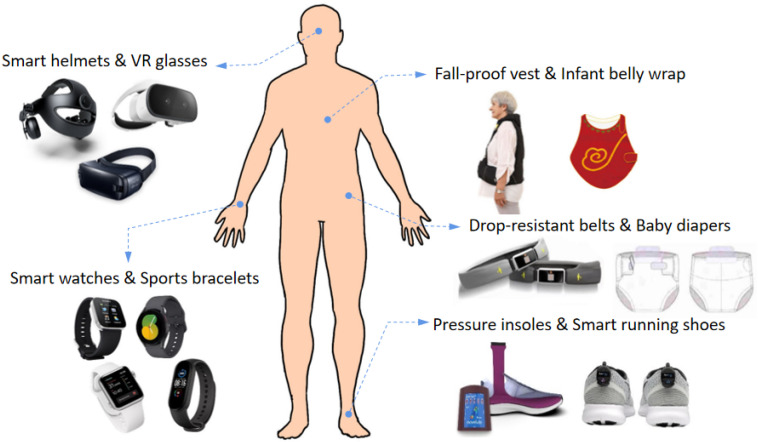
Miniaturized wearable forms.

**Figure 14:**
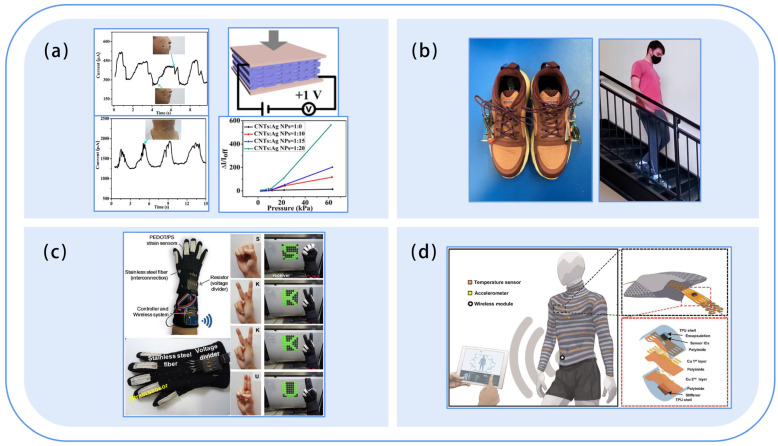
Flexible wearable forms. (**a**) SMD. Reproduced with permission [104]. Copyright 2016, American Chemical Society; (**b**) Insole. Reproduced with permission [124]. Copyright 2022, MDPI; (**c**) Glove. Reproduced with permission [125]. Copyright 2017, American Chemical Society; (**d**) Clothes. Reproduced with permission [126]. Copyright 2020, John Wiley & Sons.

The structural design of wearable systems includes the form of the sensors to be worn and the placement of the sensors. The placement of the sensor module directly affects wearing comfort. As shown in Table 5, the early sensor modules were fixed in backpacks and fanny packs or directly placed in the pockets of clothing (Figure 12), which not only brought down wearing comfort, but also resulted in poor monitoring accuracy due to the lack of close and stable contact between the sensor and human body. With the miniaturization of wearable devices, smart watches, undershirts, belts, shoe pads, glasses, and other devices emerged (Figure 13). For example, Tang et al. [121] placed the temperature and humidity sensor on the left chest of the wrap belly, and Zhang [122] placed the sensor on the waist of the diaper. YiDaiBao Company [123] designed the anti-fall undershirt for the elderly, in which they placed the six-axis sensor at the waist of the undershirt. Generally, the current research has mainly employed strapping ties, Velcro fixation, waistband undershirts, and other ways to integrate silicon-based sensors. Not only does the reduction in solidity affect the monitoring accuracy, but wearing comfort is also significantly reduced. Correspondingly, flexible sensors are mainly worn in patches, insoles, gloves, and clothing (Figure 14). Patch-type flexible sensors can be directly attached to the skin surface and are usually used for monitoring sleep conditions, breathing, and delicate movements. Zhang et al. [104] adopted two flexible sensors (Figure 14a) to monitor the muscle movement of the cheeks and throat, respectively. Insole-type flexible sensors were directly integrated with the insoles and are often used for monitoring human gait. For example, Anderson et al. [124] used a plantar pressure sensor system (Figure 14b) to monitor 13 common human movements. A glove-type flexible sensor is dedicated to hand posture monitoring and can recognize finger and palm movements. For instance, Eom et al. [125] embedded the five PEDOT/PS strain sensors in the fingertip area of the glove (Figure 14c) and interpreted sign language through different gestures. The clothing-type flexible sensors are integrated with apparel such as myoelectric garments, tights, or kinetic suits. Usually, they were placed at the collar, forehead, back, abdomen, or cuffs. The pressure of the apparel contributed to the close contact of flexible sensors with the human body. Wicaksono et al. [126] designed a customized e-textile comfort suit (Figure 14d), with the sensor elements attached to the striped textile channels of the garment. In general, we should comprehensively consider wearable comfort, detection accuracy, and safety of the sensors when designing the wearing form and the mounting position of wearable systems. Although material safety is controversial, flexible sensors are softer and more comfortable than traditional silicon-based sensors.

A summary of existing studies shows that wearable systems for human gross motion monitoring can be divided into four categories according to the application scenarios: gross motion posture recognition for adults, human rehabilitation monitoring, daily activity monitoring for the elderly, and daily activity monitoring for infants and children. As can be seen from Table 5, the installation location of sensors is associated with application scenarios. For adult gross motion monitoring, the sensors are commonly installed on the wrist, ankle, abdomen, and thigh to identify posture changes. Their monitoring indicators include the angle, direction, and acceleration of limbs and joints. Some scholars prefer to install the sensors at the wrist and ankle, while others choose to place them near the elbow and knee joints. For rehabilitation monitoring, the mounting position of the sensor changes with the desired monitoring site. Monitoring diseases related to human gait tends to mount the sensor at the ankle, thigh, and calf. In contrast, when monitoring the daily activities of rehabilitated patients, the chest, thigh, calf, upper arm, and forearm are chosen as the placement positions of sensors. In daily activity monitoring of the elderly, studies focused on fall detection. Hence, sensors are installed on the waist, abdomen, knee, and wrist. With consideration of physiological development and other factors, most research on infant activity monitoring placed sensors on the limbs, wrists, and toys.

## 6. Conclusion and Future Prospects

**Figure 15:**
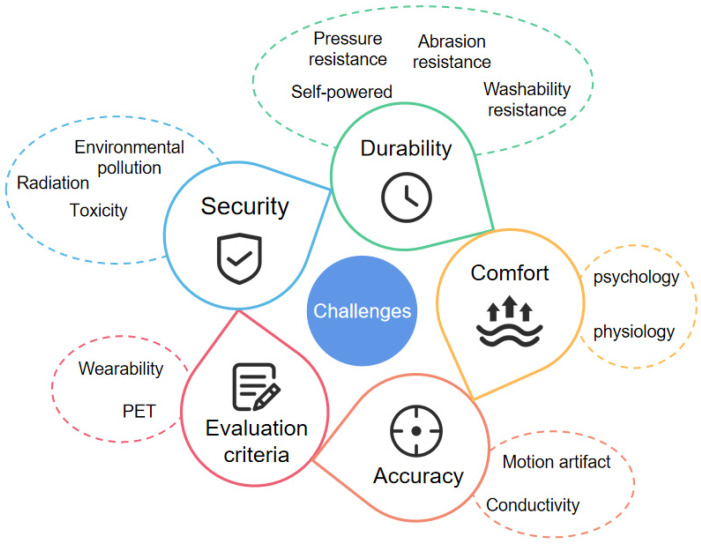
Challenges of human motion posture monitoring.

This paper provides a comprehensive and systematic review of the current research status of wearable sensors in human motion monitoring, focusing on the monitoring index system, sensor types, and system design. In most research, acceleration, inertia, tilt angle, strain, and pressure sensors are employed for real-time human motion detection. These sensors are used to recognize the changes in angle, direction, and speed of joints and limbs. They are usually placed in the appropriate positions of the upper limbs, lower limbs, lumbar spine, back, etc. The accuracy of the current motion monitoring system can reach 90%, which can realize accurate motion posture monitoring. Further, the sensors can be integrated into fabrics to wear on the body with high real-time recognition accuracy. The silicon-based sensor system is becoming more mature and is now widely used in sports monitoring. However, most of its wearing forms are very similar. Its wearing comfort also needs to be improved, and motion artifacts are still one of the biggest challenges in wearable sports monitoring. Correspondingly, flexible sensors have been extensively researched. They could recognize large deformations in human joints, and some have begun to be commercialized. There are still many technical, industrial, and market limitations. The problems are summarized and specified as follows (Figure 15).

### 6.1. Security

Wearable electronic products use many electronic components. As a result, there may be risks of charge leakage and electronic radiation, especially for flexible electronic fabrics. Besides, fabrics will be worn on the skin in a long-term manner. Thus, the biosafety of substrates and conductive materials becomes more critical. Among the widely used carbon-based materials, carbon nanotubes are more hazardous in terms of toxicity and environmental pollution [11]. In addition, as wearable electronic products need to collect a large amount of data, including physiological data, environmental data, geographic location, lifestyle habits, and other private information, the disclosure of personal information will lead to severe consequences.

### 6.2. Durability

The high power consumption of sensors, data processing, and analytic hardware limits the durability of wearable device operation [139]. Reducing the sensor accuracy or switching the standby and wake-up states of the wearable system can alleviate this problem. Alternatively, some scholars have developed innovative self-powered devices [140,141]. They convert solar energy, human movement mechanical energy, etc. into electrical energy. However, the energy supply efficiency of flexible electronic devices in current research is very low and cannot meet real-time wearable needs. In addition, to ensure the durability and monitoring accuracy of smart clothing after multiple washes, intelligent wearable devices should have the capability of wearing resistance, pressure resistance, bending resistance, and washability.

### 6.3. Comfort

With the rising requirements of motion monitoring, sensor networks are becoming increasingly complex to monitor multiple body parts. This has posed a significant challenge to wearing comfort. Currently, hardware sensor-integrated wearable systems come in the same form and are unsuitable for particular groups, such as the elderly and infants. Intelligent clothing systems integrated with flexible sensors, especially textile sensors, are promising to greatly enhance wearing comfort. However, the high costs and testing accuracy limit their large-scale commercial production and application. In addition, wearing comfort has both physiological and psychological aspects. Thus, it is needed not only to ensure the physiological comfort of wearing it, but also to meet the psychological needs of users. Wearing devices should have a certain sense of aesthetics, and color matching should be appropriate.

### 6.4. Accuracy

As a wearable device for sports monitoring, accuracy is of utmost importance. The limitations of hardware sensors involve soft tissue artifacts, sensor movement, and the relative movement of bones, among others. In comparison, the flexible sensor is more skin-friendly and more accurate. It can effectively reduce the error caused by the movement of the sensor. The integration of nanomaterials into research has dramatically increased the conductivity of flexible sensors [142]. However, its elasticity still needs to be improved. The sensor may bend, stretch, or be subjected to normal pressure during motion monitoring, which may affect the accuracy of the measurement. In addition, flexible sensors on textile and thin film substrates generally suffer from noticeable mechanical hysteresis and relaxation due to the use of polymeric materials [143,144,145]. This requires a more detailed study at the sensor design and algorithm level.

### 6.5. Evaluation Criteria

Due to the differences in functions and characteristics of flexible and traditional electronic devices, the industrial standards of wearable products are not universal. Flexible devices should not only meet the electronic requirements, but also meet the wearing demands, including dimensional stability, breathability, appearance, feel, etc. They should withstand large deformation, repeated strain, bending, and pressure [131]. Furthermore, they must have washing, perspiration, bending, abrasion resistance, etc. These requirements need a unified standard for evaluation. Nonetheless, the development of flexible electronic evaluation systems in different countries and regions is still in the embryonic stage. The relevant research standards are very rare, and no universal systematic evaluation system has been established.

In the future, sensor-based human motion monitoring devices will be more accurate and comprehensive. Miniaturized, lightweight, energy-efficient, and unobtrusive wearable devices will be the trend in human posture monitoring. Textiles and clothing, as favorable carriers for sensor system integration, can achieve proper comfort in wearable monitoring systems if the flexibility of hardware sensors, wires, and batteries can be achieved. Then it is possible to fully integrate them with garments and accurately arrange the three-dimensional coordinates of the sensors. The next-generation wearable sensing technology for human movement monitoring will have bright future prospects, from auxiliary devices for telemedicine to achieve the whole process of monitoring the growth and development of infants and children to scenarios of personal sports and fitness guidance and posture correction, among others.

## Figures and Tables

**Table 1:** Movement amplitudes of each part of the human body.

Body Parts	Joint	Direction of Motion	Action	
			No.	Angle/(°)
Head	Vertebral column	Levorotation	HE1	55
		Dextrorotation	HE2	55
		Flexion	HE3	40
		Hyperextension	HE4	50
		Right lateral flexion	HE5	40
		Left lateral flexion	HE6	40
Trunk	Vertebral column	Flexion	TR1	100
		Hyperextension	TR2	50
		Left lateral flexion	TR3	50
		Right lateral flexion	TR4	50
Hands	Wrist joints	Extension	HA1	65
		Flexion	HA2	75
		Adduction	HA3	30
		Abduction	HA4	15
		External rotation	HA5	90
		Internal rotation	HA6	80
Forearms	Elbow joints	Flexion	FO1	145
		Extension	FO2	0
Calves	Knee joints	Flexion	C1	135
		Extension	C2	0
Upper arms	Shoulder joints	Flexion	U1	180
		Extension	U2	45
		Horizontal abduction	U3	40
		Horizontal adduction	U4	140
		Abduction	U5	180
		Adduction	U6	45
Thighs	Hip joint	Flexion	TH1	120
		Extension	TH2	45
		Abduction	TH3	45
		Adduction	TH4	40
		Internal rotation	TH5	35
		External rotation	TH6	30
Feet	Ankle joints	Dorsiflexion	FE1	20
		Plantar flexion	FE2	45
		Adduction	FE3	45
		Abduction	FE4	50

**Table 2:** Accelerometers for human motion and posture monitoring.

Model	Company	Size (Length × Width × Thickness) (mm × mm × mm)	Range of Measurement (g)	Sampling Rate (Hz)	Application Areas
ADXL330 Triaxial Accelerometer [51]	ADI, Norwood, MA, USA	4 × 4 × 1.45	±3	100	Waist and thighs
ADXL345 Triaxial Accelerometer [52,53,54,55,56]	ADI, Norwood, MA, USA	3 × 5 × 1	±2	50, 100	Abdomen, upper limbs, thighs, and calves
ADXL362 Accelerometer [57]	ADI, Norwood, MA, USA	3 × 3.25 × 1	±2	100, 400	Waist, thighs, and calves
MMA7260 3-axis Accelerometer [46,58]	Freescale, Austin, TX, USA	6 × 6 × 1.45	±1.5	\	Chest pockets, waist, left and right front pant pockets, back pant pockets, and the inside of the jacket
LIS3DH Triaxial Accelerometer [59]	STMicroelectronics, Geneva, Switzerland	3 × 3 × 1	±2	100	Android phones
MMA8453 3-axis Accelerometer [60]	Freescale, Austin, TX, USA	3 × 3 × 1	±2	1.56–800	Trunk

(Notes: all the commercialized accelerometers above are triaxial).

**Table 3:** Gyroscopes for human motion and posture monitoring.

Model	Company	Number of Axes	Size (Length × Width × Thickness) (mm × mm × mm)	Range of Measurement (dps)	Sampling Rate (Hz)	Application Area
L3G4200D	STMicroelectronics, Geneva, Switzerland [61]	3	4 × 4 × 1	±2000	100, 800	Forearms and upper arms
IMUZ	ZMP, Tokyo, Japan [62]	6	21 × 21 × 22	±500	100	Waist
3DM-GX3-25	Honeywell, Morris, NJ, USA [63]	9	44 × 25 × 11	±300	100	Backpack handle
ISM330DHCX	STMicroelectronics, Geneva, Switzerland [64]	6	2.5 × 3 × 0.83	±4000	12.5–6700	Upper arms, forearms, hands, and trunk

**Table 4:** Inertial sensors for human motion and posture monitoring.

Model	Company	Size (Length × Width × Thickness) (mm × mm × mm)	Application Areas
MPU6050 3-axis acceleration Gauges and Gyroscopes [54,65,66]	InvenSense, Sunnyvale, CA, USA	4 × 4 × 0.9	Chest, abdomen, wrists, thighs, calves, and feet
Flow-MIMU 3-axis accelerometer and gyroscope [67]	\	\	Wrists and calves
YD122 Inertial Sensor [6]	Hundred Years Xukang Medical Equipment Co. Chengdu, Sichuan, China	\	Chest, thighs, calves, upper arms, and forearms
MPU9250 Inertial Sensor [68]	InvenSense, Sunnyvale, CA, USA	3 × 3 × 1	Chest, thighs, and wrists
BMI160 Inertial Sensor [43]	Bosch, Stuttgart, Germany	2.5 × 3 × 0.8	Smart Watch
MPU6500 Inertial Sensor [69]	InvenSense, Sunnyvale, CA, USA	3 × 3 × 0.9	Thighs, wrists, and ankle joints

(Note: all the commercialized inertial sensors above are nine-axis).

**Table 5:** Wearable sensor system design for human motion posture monitoring.

Application Scenarios	Monitoring Objectives	Hardware Design		System Architecture Design	
		Sensors and Numbers	Monitoring Accuracy	Location	Connections
Sports detection and monitoring of adults	For the measurement of human posture and movement [127]	A microelectromechanical sensor (consisting of an accelerometer and a magnetometer)	\	On two connected limbs	\
	Upper limb movement recognition [53]	One ADXL345 triaxial accelerometer	\	Upper limb (left or right limb)	Strap bondage
	Monitoring human trunk movements [128]	A sensing system consisting of an accelerometer, gyroscope, and magnetometer	The root mean square error is 1.81 ± 0.77°	Dorsal T9 to T10 at the thoracic vertebrae	Sticky
	Monitoring the body’s motion posture during high dynamic motion [67]	Two Flow-MIMU motion capture devices (consisting of a three-axis accelerometer and a three-axis gyroscope)	Much lower drift rate in limb roll angle and pitch angle	Wrist and calf	Strap bondage
	Human motion detection and recognition [45]	A triaxial accelerometer	Recognition accuracy of 85.17%	Wrist	\
	Identify the seven movements of the human body in daily life [51]	Two sensor modules (tri-axis acceleration sensing) (composed of ADXL330 and a microgyroscope)	Recognition accuracy reached 91.22%	On the waist and thigh	Upper thigh straps, waist belt
	Monitoring exoskeleton gait [54]	5 × MPU-6050 type 6-axis MEMS inertial sensors	\	Abdomen, upper one-third of the thighs and lower one-third of the calves	Waist belt, leg velcro straps
	Characterize human motion posture [42]	Four BWT901-type nine-axis attitude sensors (gyroscope, accelerometer, geomagnetic field sensor composition)	\	Both wrists and ankles	Taping
	Human posture monitoring [57]	Five MEMS sensors (consisting of an accelerometer, magnetometer, and barometer)	\	Waist and left and right forearms near the wrist joint, left and right calves near the ankle joint	Belts and straps
	Monitoring of joint and micro-muscle movements [104]	Three flexible strain sensors	\	Knees and arms	Film adhesion
	Health Diagnosis [129]	A flexible pressure sensor	High sensitivity (10.1(kPa^−1^))	Back of hand or index finger	Medical tape bonding
	Monitoring foot conditions during daily activities [113]	Six textile sensors (three on the front foot and three on the heel)	Error less than 1%	Soles of the feet	Shoe insoles
	Monitoring calf muscles in daily exercise [115]	8 × 16 pressure sensing array	\	Wrists	Smart sports bracelet
Monitoring of human rehabilitation therapy	Remote access to the daily exercise status of rehab patients [6]	9 YD122 inertial sensors	The joint mobility error is within 5°, and the system delay is within 0.5 s	Chest, left and right outer thighs, left and right outer calves, left and right outer upper arms, and left and right outer forearms	Soft Tape
	Monitoring the progress of rehabilitation after TKR (total knee replacement) [35]	Two MPU6050 inertial measurement units (IMUs)	Accuracy of 96.16~98.09% at different DK speeds	Thighs and ankles	Velcro straps
	Tonic-clonic epilepsy detection [31]	An accelerometer	7 out of 8 seizures detected	Wrist	Watches
	Recovery of daily activities during hospitalization after major surgery [130]	An accelerometer	\	Ankle joint	Velcro straps on the outside of the socks
	Effect of gait training on walking ability in patients with gait disorders due to cerebral palsy [131]	One inertial sensor	\	Eyeglass frames	Eyeglasses
Daily activity monitoring of the elderly	Fall prediction for the elderly [65]	A tactile force sensor, three-axis accelerometer, and gyroscope combination	Accuracy rate up to 95%	Upper Trunk	\
	Continuous long-term activity monitoring for the elderly [46]	A triaxial accelerometer	Average recognition accuracy of 94.4%	Any pocket	Clothing
	Home care and fall monitoring for the elderly [30]	Three triaxial accelerometers	Average Accuracy of 96.54%	Lower back, wrists, and knees	\
	Fall detection warning for the elderly [132]	Three joint angle sensors	100% accuracy for fast running and squatting; 98.7% and 97.2% accuracy for front side and side falls	Knee, lower back	Smart clothing
Daily activity monitoring of children	Ecological assessment of motor performance in infants aged 12–36 months [133]	Magnetic inertial platform with three sensors: two wired magnetic inertial sensors, one wireless Bluetooth magnetic motor inertial module	The technology platform will be implemented in the future	Wrist, toys	Small toys inside and soft straps
	Quantifying daily leg movements for infants [134]	Two inertial sensors	Can measure 10 ms of no motion between two motions	Ankle joints	Fixed in stockings
	The number of times the infant sucks at meals [135]	A piezoelectric jaw motion sensor	Average error rate of −9.7%	Under the ear, behind the chin	Duct tape fixing
	Measuring the joint angle during the infant’s kicking [136]	Four joint-angle sensors	\	On the hips and knees	\
	Assessment of trunk posture and arm movements in infants [137]	Five IMUs (Inertial Measurement Units)	Error below 10%	Chest, forearms, upper arms	Undershirts and straps
	Characterize the dynamics of the infant’s limbs [138]	Four accelerometers	\	Four limbs	\

## Data Availability

No new data were created or analyzed in this study. Data sharing is not applicable to this article.
